# ReCodLiver0.9: Overcoming Challenges in Genome-Scale Metabolic Reconstruction of a Non-model Species

**DOI:** 10.3389/fmolb.2020.591406

**Published:** 2020-11-26

**Authors:** Eileen Marie Hanna, Xiaokang Zhang, Marta Eide, Shirin Fallahi, Tomasz Furmanek, Fekadu Yadetie, Daniel Craig Zielinski, Anders Goksøyr, Inge Jonassen

**Affiliations:** ^1^Department of Computer Science and Mathematics, Lebanese American University, Byblos, Lebanon; ^2^Computational Biology Unit, Department of Informatics, University of Bergen, Bergen, Norway; ^3^Department of Biological Sciences, University of Bergen, Bergen, Norway; ^4^Department of Mathematics, University of Bergen, Bergen, Norway; ^5^Institute of Marine Research, Bergen, Norway; ^6^Department of Bioengineering, University of California, San Diego, La Jolla, CA, United States

**Keywords:** genome-scale metabolic reconstruction, Atlantic cod, less-annotated species, model curation, environmental toxicology

## Abstract

The availability of genome sequences, annotations, and knowledge of the biochemistry underlying metabolic transformations has led to the generation of metabolic network reconstructions for a wide range of organisms in bacteria, archaea, and eukaryotes. When modeled using mathematical representations, a reconstruction can simulate underlying genotype-phenotype relationships. Accordingly, genome-scale metabolic models (GEMs) can be used to predict the response of organisms to genetic and environmental variations. A bottom-up reconstruction procedure typically starts by generating a draft model from existing annotation data on a target organism. For model species, this part of the process can be straightforward, due to the abundant organism-specific biochemical data. However, the process becomes complicated for non-model less-annotated species. In this paper, we present a draft liver reconstruction, ReCodLiver0.9, of Atlantic cod (*Gadus morhua*), a non-model teleost fish, as a practicable guide for cases with comparably few resources. Although the reconstruction is considered a draft version, we show that it already has utility in elucidating metabolic response mechanisms to environmental toxicants by mapping gene expression data of exposure experiments to the resulting model.

## 1. Introduction

Induced by the availability of next-generation DNA sequencing as well as high-throughput genomic, proteomic, and metabolomic data, genome-scale metabolic models (GEMs) have become fundamental tools in the systems biology of metabolism. To develop a GEM, metabolic genes and their product enzymes are assembled into a network of metabolic reactions carried out by an organism (Palsson, 2006). Such networks can be used as a platform to answer relevant biological questions, by transforming biochemical knowledge into a mathematical format and computing appropriate physiological states (Orth et al., 2010; Schellenberger et al., 2011). Using Boolean logic representation, gene-protein-reaction (GPR) associations depict direct connections between genotype and metabolic capability (Nielsen, 2017). GEMs have a wide scope of applications, among which are the contextualization of omics data, hypothesis-driven discovery, support for metabolic engineering, modeling interactions among cells and organisms, drug targeting, and prediction of enzyme functions (Oberhardt et al., 2009; Gu et al., 2019).

A comprehensive bottom-up protocol to generate high-quality reconstructions was presented a decade ago (Thiele and Palsson, 2010). It consists of four main phases presented as an exhaustive list of steps. First, a draft reconstruction is generated based on the genome annotation of a target organism and available information in biochemical databases, usually in an automated manner. Noticeably, the quality of the draft model is highly impacted by the quality of the genome annotation, in addition to the abundance of data on the organism in public repositories. The draft is then subject to manual refinement that revises and curates all gene and reaction entries. Next, the reconstruction can be converted into mathematical format which is thereafter analyzed for gap filling and tested for stoichiometric balance. Several computational tools were developed to cover this process with automated functionalities (Gu et al., 2019). They primarily vary in programming languages, linked databases, input template models, and supported target organisms.

Marine organisms, including fish, represent important resources for human societies, and will be essential components of a growing blue bioeconomy. As we are entering the UN Decade of Ocean Science for Sustainable Development (Visbeck, 2018; Ryabinin et al., 2019), understanding and predicting the effects of environmental stressors, including xenobiotic exposures, on marine species will be one of the main challenges. In this context, metabolic reconstructions can prove very useful. Notwithstanding, when it comes to teleost GEMs, only two models are presented in the literature, MetaFishNet (Li et al., 2010) and ZebraGEM (Bekaert, 2012; van Steijn et al., 2019). MetaFishNet covers five fish species which makes it challenging to analyze organism-specific data using this model. In addition, subsystems in this GEM are not interconnected or compartmentalized. The first version of the ZebraGEM, a zebrafish GEM, did not contain GPR associations. A very recent version, ZebraGEM 2.0 (van Steijn et al., 2019), now includes those associations, with standardized component names, and expanded reactions list.

The Atlantic cod (*Gadus morhua*) is a key species in the North Atlantic marine ecosystem. Its genome was first published in 2011 (Star et al., 2011) and has been followed up by more extensive genome sequencing, enabling novel genomic and transcriptomic assemblies (Torresen et al., 2017) as well as a Trinity assembly (Zhang et al., 2020) and a very recent assembly gadMor3.0 (GenBank assembly accession: GCA_902167405). These resources allow extensive gene mapping and bioinformatic analysis, such as the identification of the gene complement of the Atlantic cod cytochrome P450 superfamily (Karlsen et al., 2012) or the total chemical defensome (Zhang et al., 2019). Due to its habitat near offshore installations as well as coastal industries and communities, the Atlantic cod serves as a relevant model species for studying the effects of and responses to anthropogenic activities (Dale et al., 2019). In addition, the lipid-rich liver of cod serves as a magnet for lipophilic environmental pollutants, increasing the bioaccumulation within each fish with age and the biomagnification of compounds through the food chain.

The Atlantic cod has lost the MHC II and related genes of the adaptive immune system (Star et al., 2011). Furthermore, we have recently shown that cod, together with several other teleost fish, have lost the pregnane x receptor (*pxr, nr1i2*) gene from their genomes (Eide et al., 2018). This xenosensor is activated by a wide range of drugs and pollutants, and regulates the transcription of genes involved in all phases of the xenobiotic and steroid biotransformation (Kliewer et al., 2002). PXR also mediates regulation of hepatic energy metabolism in humans, by inducing lipogenesis and inhibiting fatty acid beta-oxidation (Wada et al., 2009). The absence of a *pxr* gene thus leads to questions regarding how the Atlantic cod responds to environmental contaminants that bind this receptor in other species. Results from an *in vivo* exposure study, where cod was injected the legacy polychlorinated biphenyl congener PCB153, a compound known to bind PXR in other species, showed indications that lipid metabolism was affected (Yadetie et al., 2017). In view of these findings, an ensuing effort was initiated to generate a metabolic reconstruction of Atlantic cod liver, with a focus on lipid and xenobiotic metabolism pathways. In this paper, we present and discuss our metabolic reconstruction progress in terms of steps, methods, choice of tools, and challenges.

## 2. Methods

### Selecting the Appropriate Tool

Many computational tools have been developed to help automate steps of the GEM reconstruction process which is usually laborious and time-consuming (Thiele and Palsson, 2010). The choice of tool for our reconstruction work on Atlantic cod was predominantly narrowed down by the fact that this is a non-model and less-annotated organism. *De novo* reconstruction based on metabolic databases do not apply in such cases, but rather GEMs of related scope needs to serve as input. Another important feature for us was the tool's support for eukaryote modeling. In view of these constraints, the Reconstruction, Analysis, and Visualization of Metabolic Networks (RAVEN 2.0) MATLAB toolbox (Wang et al., 2018) was selected. It is used for semi-automated draft model reconstruction and provides curation, simulation, and constraint-based capabilities that are compatible with the COBRA toolbox. RAVEN has been used to generate GEMs for several organisms including bacteria, archaea, parasites, fungi, and various human tissues. We also note that the toolbox allows *de novo* draft reconstruction based on metabolic pathway databases, namely KEGG and MetaCyc, although that it is not applicable to our case study. Most importantly, RAVEN can generate draft models for a target organism based on protein homology, using existing high-quality GEMs of organisms at an appropriate evolutionary distance. Two main functions are applied in this process. getBlast or getBlastFromExcel construct a structure including homology measurements between the target organism and the template organisms. Then, getModelFromHomology uses this structure along with existing template GEMs to create a draft model containing reactions associated with orthologous genes. The subsequent draft is then subject to curation and refinement. The RAVEN toolbox includes several functions that are implemented to support subsequent steps.

We also highlight a few approaches. CarveME (Machado et al., 2018) uses a top-down approach, based on Python command-line, to convert curated reactions from the BiGG database into organism-specific GEMs. AutoKEGGRec (Karlsen et al., 2018) is a novel tool which is based on the KEGG pathways database (Aoki and Kanehisa, 2005) and is fully integrated with the COBRA toolbox (Schellenberger et al., 2011). AuReMe (Aite et al., 2018) presents a customized platform and pipelines to generate GEMs while preserving metadata and ensuring model traceability. It is also linked to the BiGG knowledgebase (Duarte et al., 2007) and the MetaCyc database (Caspi et al., 2013). For a thorough review of GEM reconstruction tools, we refer to the recent paper by Mendoza et al. (2019). The tools available for non-model species reconstructions (Henry et al., 2010; Boele et al., 2012; Hanemaaijer et al., 2017; Machado et al., 2018; Wang et al., 2018) are listed in Supplementary Table S1.

### Choosing the Reconstruction Template

With the Atlantic cod liver as our reconstruction scope, we surveyed the literature for a relevant GEM that could be used as an input template to the RAVEN 2.0 toolbox. In the absence of tissue-specific models for fish species and the availability of a human liver GEM that greatly focuses on lipid metabolism, a primary trade-off was between using a generic template from a phylogenetically closer species, i.e., zebrafish, or using a liver-specific template from human that falls within our reconstruction scope. The iHepatocytes2322 (Mardinoglu et al., 2014) consensus GEM was our template of choice. The reactions and associated proteins included in iHepatocytes2322 were assigned into eight compartments. More details can be found in the original publication (Mardinoglu et al., 2014).

Of the two available fish GEMs, the MetaFishNet (Li et al., 2010) involves five fish species: zebrafish (*Danio rerio*), medaka (*Oryzias latipes*), Takifugu (*Takifugu rubripes*), Tetraodon (*Tetraodon nigroviridis*), and stickleback (*Gasterosteus aculeatus*). Here, cDNA sequences from those species were analyzed and the corresponding list of metabolic genes was formed based on gene ontology. This list was then used to identify enzymes based on their orthology to human genes. Metabolic reactions were integrated from EHMN, the human metabolic network at UCSD (BiGG) (Duarte et al., 2007), and the D. rerio metabolic network from KEGG (Aoki and Kanehisa, 2005). The other fish model is ZebraGEM (Bekaert, 2012). It is a comprehensive GEM based on the literature and D. rerio biochemical data collected from various resources. This model is manually curated with gaps extensively checked and filled. An updated version, ZebraGEM 2.0, now includes the GPR associations and essential reactions, which were not previously covered in the first version. Both versions simplify the description of lipid metabolism processes by mainly discarding reactions specific to fatty acids. Therefore, essential pathways in our reconstruction scope were excluded from ZebraGEM, which makes it unfit to our case study.

Initially, the global reconstruction of the human metabolic network, Recon 1, was published in 2007 (Duarte et al., 2007). Several network and supporting information updates have followed. A recent Recon 3D version includes a thorough cataloge of GPR associations in addition to structural information on metabolites and enzymes (Brunk et al., 2018). This version also comprises a three-dimensional metabolite and protein structure data which allow integrated analyses of metabolic functions in humans. The Edinburgh Human Metabolic Network (EHMN) was presented concurrently to Recon 1 (Ma et al., 2007). The Human Metabolic Reaction (HMR) database (Agren et al., 2012) was later generated by integrating Recon 1 and EHMN with biochemical reactions from KEGG (Aoki and Kanehisa, 2005) and MetaCyc (Caspi et al., 2013) databases. An updated version (HMR 2.0) with emphasis on lipid metabolism was later introduced (Mardinoglu et al., 2014). Several subsequent tissue- and context-specific GEMs were released. They include iAdipocyte1809, iMyocytes2419, and CardioNet (Mardinoglu et al., 2013; Geng and Nielsen, 2017). We emphasize the consensus GEM for hepatocytes, iHepatocytes2322, which was assembled from proteomics data and previously published liver models. It is a consensus hepatocyte GEM that is mainly generated from proteomics data of several existing liver models, namely HepatoNet1 (Gille et al., 2010), iLJ1046 (Jerby et al., 2010), iAB676 (Bordbar et al., 2011), and iHepatocyte1154 (Agren et al., 2012). It is well-annotated as it includes all the protein-coding genes and reactions in those liver models. Notably, and in alignment with our GEM context, iHepatocytes2322 extensively covers lipid metabolism functions, such as the uptake of remnants of lipoproteins, the formation and degradation of lipid droplets, and secretion of synthesized lipoproteins, as well as xenobiotic biotransformation. It consists of 2,322 genes, 5,686 metabolites, and 7,930 reactions, separated into eight different compartments based on the HMR 2.0 database. Although the template is not fish-specific, the important subcellular functions and mechanisms within the compartments are conserved through evolution, particularly among fish and humans (Schlegel and Stainier, 2007; Li et al., 2010). Indeed, Zebrafish is widely used model to study human metabolic diseases (Schlegel and Stainier, 2007; Seth et al., 2013; Gut et al., 2017). However, there are differences in fat storage sites between Atlantic cod and humans, the molecular bases of which are not known. In Atlantic cod and other lean fish fat is stored mainly in the liver, whereas in humans it is stored in adipose tissues. In fact, cod liver normally contains 50–60% of fat (Li et al., 1986) that is stored in peroxisomes and cytosolic lipid droplets, and these subcellular compartments are also included in iHepatocytes2322.

### Generating an Atlantic Cod Liver Draft Model

As described, we used the RAVEN 2.0 toolbox to generate a draft metabolic reconstruction of the Atlantic cod liver, based on protein homology, and using iHepatocytes2322 as our input template.

**Figure 1:**
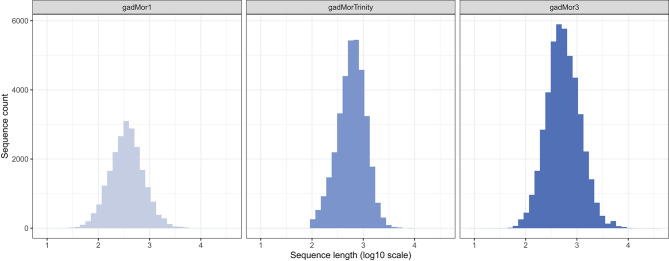
The length distribution of the peptide sequences from three assemblies of Atlantic cod.

In addition to the template model as input, RAVEN 2.0 requires two FASTA files including the peptide sequences of the target and the template organisms. The literature and public repositories contain four main versions of Atlantic cod genome annotations. The first assembly, gadMor1, was published in 2011 (Star et al., 2011). An updated version, gadMor2, with extensive genome sequencing enabling novel genomic and transcriptomics assemblies followed in 2017 (Torresen et al., 2017). Those two versions along with multiple RNA-seq data from multiple tissues and developmental stages were used to create gadMorTrinity (Zhang et al., 2020) which includes less-fragmented and more comprehensive sequences. gadMor3.0 (GenBank assembly accession: GCA_902167405), here gadMor3, is a recently published chromosome-level reference assembly generated using long-read sequencing technology. The length distribution of the peptide sequences from these three assemblies are shown in Figure 1. We used gadMor1, gadMorTrinity, and gadMor3 to examine the potential improvement that these annotations could bring in the context of metabolic reconstructions. A separate set of draft models was generated from each assembly.

**Figure 2:**
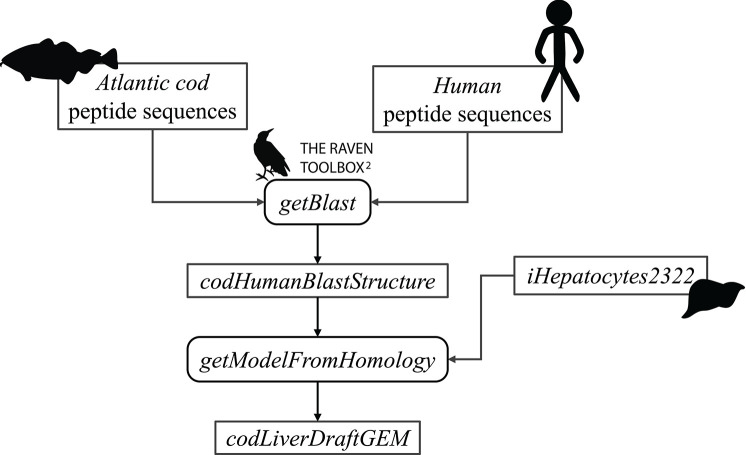
Schematic overview of the reconstruction of an Atlantic cod liver draft model.

First, the FASTA files were used as input to the getBlast function. A BLAST structure, was created from the reciprocal BLAST analyses between the target and the template organism, using the BLASTp algorithm (Altschul et al., 1990). Next, this structure along with the template model were input to the getModelFromHomology function to generate a draft cod GEM by extracting reactions involving cod genes that are orthologous to human genes in iHepatocytes2322. Figure 2 shows an overview of the draft model reconstruction process. The process can be tuned based on different input parameters.

We used the RAVEN 2.0 toolbox to generate several draft models based on different strictness parameters and Atlantic cod genome annotations. The strictness parameter specifies which reactions are included in the draft model, mainly based on how genes from the template GEM organism and from the target organism are mapped against each other. When strictness is equal to 1 or 2, gene mapping is done for all pairs based on BLASTp score threshold, in both or in one direction, respectively. Alternatively, when strictness is set to 3, all BLASTp results are checked and only the ones with the lowest *E*-value are kept for all gene pairs in each direction separately. Then, target genes are mapped to template genes for all pairs which have acceptable BLASTp results, in both directions. In addition, maxE, minLen, and minIde are adjustable thresholds for the maximum E-value, the minimum alignment length, and the minimum identity acceptable in the gene mapping process. Their respective default values are 10^−30^, 200, and 40%.

## 3. Results

**Figure 3:**
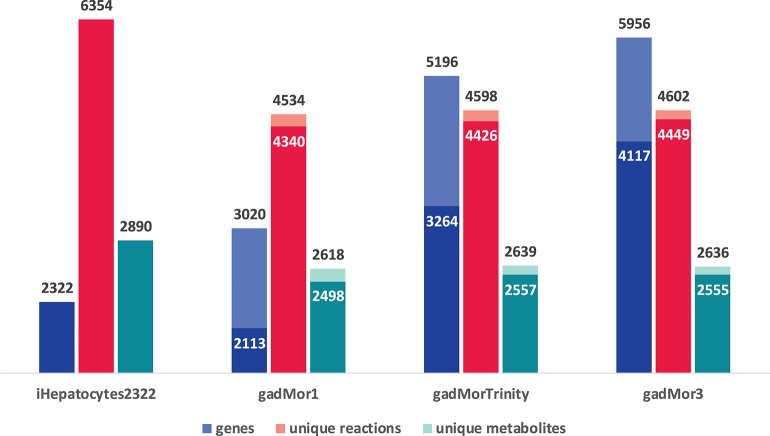
Model statistics of iHepatocytes2322 compared to draft reconstructions of Atlantic cod liver, using different annotations as reference. Dark color indicates strictness of s3 (one to one) and light color indicates strictness of s1 (one to many).

The graph in Figure 3 shows the model statistics corresponding to iHepatocytes2322 and different cod annotations, based on strictness values of 1 (s1) and 3 (s3) for one-to-many and best one-to-one mapping between human and cod genes, respectively. First, we note that s1 leads to a greater number of matched genes in the draft models. Such high number of matches can be partly attributed to the fish specific whole-genome duplication during teleost fish evolution, where fish genomes may have more than one orthologs for some human genes (Li et al., 2010; Glasauer and Neuhauss, 2014; Grimholt, 2018; Ravi and Venkatesh, 2018). However, a higher proportion of these matches are likely to be a result of splice variants and transcript assembly artifacts. Accordingly, the more annotated the cod genome, the more duplicate matches with human genes in the template are expected. In this context, the trend in the number of genes in the draft models can be attributed to the fact that gadMor1 has the lowest while gadMor3 has the highest number of annotated sequences. The number of reactions and metabolites across all resultant drafts is close for both strictness values.

**Figure 4:**
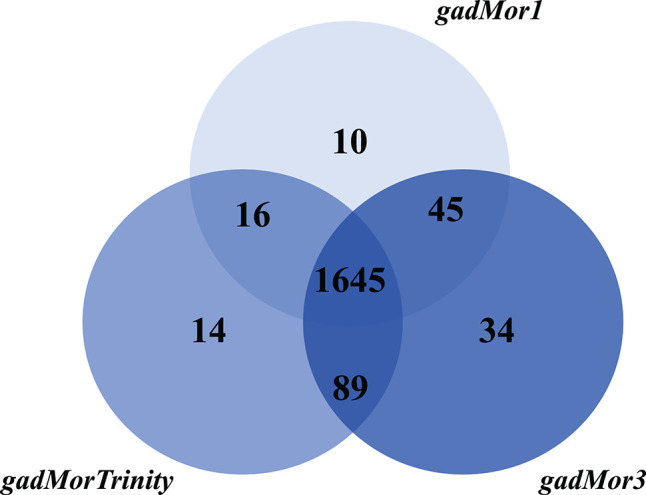
Number of genes in the Atlantic cod liver draft GEMs based on the three genome assemblies, gadMor1, gadMorTrinity, and gadMor3, when mapped to iHepatocytes2322 with strictness equal to 3.

The current version of the RAVEN toolbox adds a reaction to the draft if at least one of its catalyzing enzymes is mapped between species. Hence, the number of reactions and metabolites as produced by the printModelStats function in the toolbox does not reflect the actual gene mapping between iHepatocytes2322 and each of the draft models. One way to properly examine different cod annotation outcomes, given the known fish genome duplication, is to compare the number of mapped genes from iHepatocytes2322 with each draft, with strictness s3. But the current RAVEN only generates the mapped genes included in the draft model, we thus added this feature to the getModelFromHomology function and pulled the request to RAVEN's GitHub page (https://github.com/SysBioChalmers/RAVEN/pull/301). The Venn diagram in Figure 4 shows the output of this function. As the draft model from gadMor3 is the most complete, we used it as the baseline and included the other two as complementary sources. All three draft models were deposited in BioModels (Malik-Sheriff et al., 2020) and assigned the identifiers MODEL2010090001, MODEL2010090002, MODEL2010090003. They are also linked to the FAIRDOMHub (Wolstencroft et al., 2017) assay “Draft metabolic reconstruction model of Atlantic cod liver” (https://doi.org/10.15490/fairdomhub.1.assay.1365.2).

**Figure 5:**
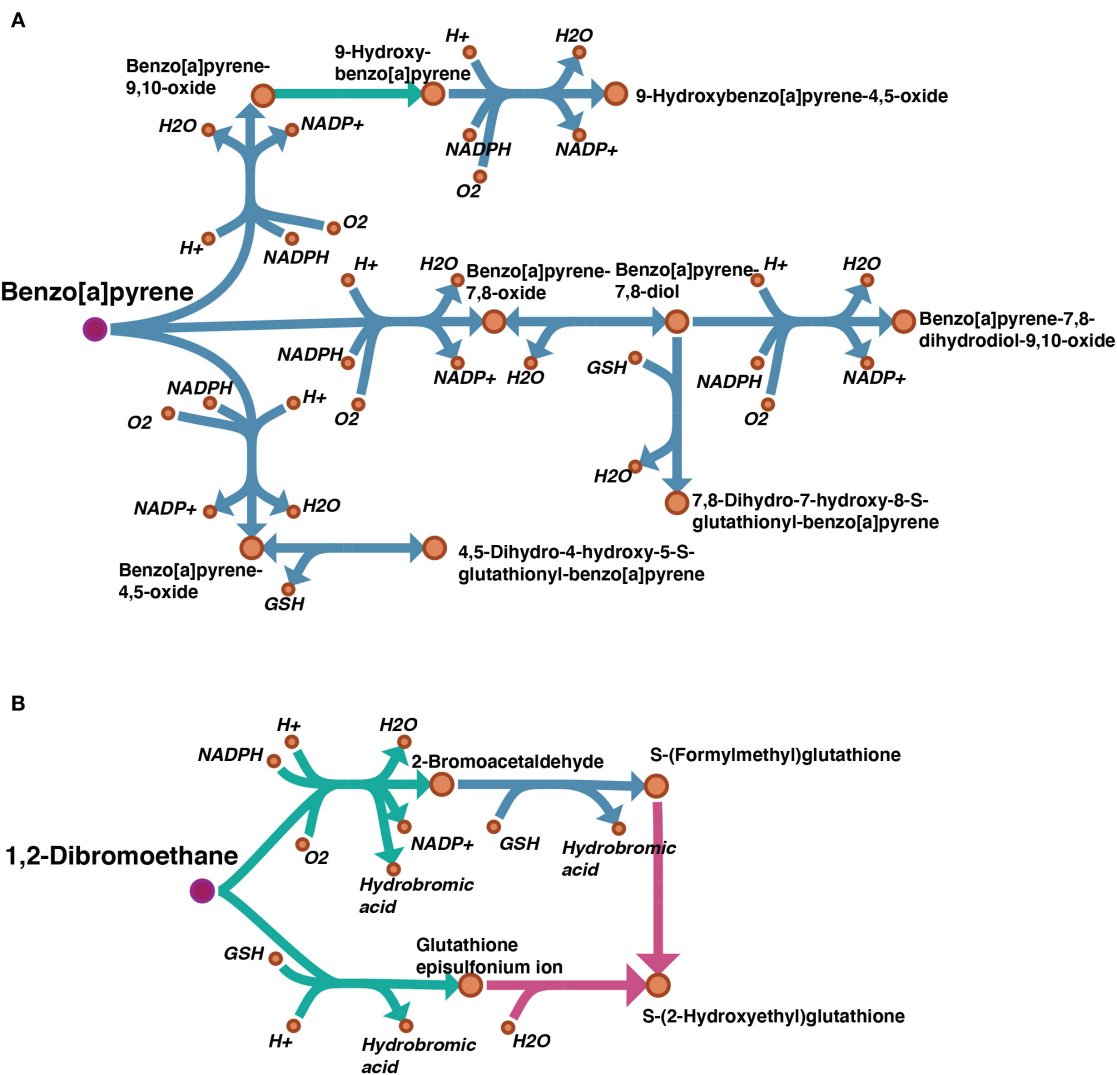
Visualization of gap filling of two pathways picked from the subsystem “Metabolism of xenobiotics by cytochrome P450”: **(A)** benzo(a)pyrene (BaP); **(B)** 1,2-Dibromoethane. The reactions existing in the auto-generated draft model are shown in blue, and the manually filled gaps are shown in green. Comparing our draft model with the same subsystem from model iHepatocytes2322, the missing reactions are highlighted in pink. The pink dots indicate the parent toxicants.

A draft reconstruction typically contains missing genes and reactions which are gaps to be filled later. In cases where a template model is used as input, such gaps result from unmapped genes, and subsequent reactions, between the template and the target organisms. In order to examine the degree of gaps in our draft model, we considered the “Metabolism of xenobiotics by cytochrome P450” subsystem which is highly relevant to our case study. A map showing the gap filling of this subsystem was visualized by Escher (King et al., 2015) and can be found on FAIRDOMHub at https://doi.org/10.15490/fairdomhub.1.datafile.3892.1. The pathways of benzo[a]pyrene (BaP) and 1,2-Dibromoethane from this subsystem are shown in Figure 5.

By manual curation, we set several criteria for gap filling: 1. by searching relevant literature, several reactions were identified as spontaneous reactions, i.e., not catalyzed by any enzyme, and included; 2. when the chemistry of the reaction made it clear that it belonged to a process catalyzed by a certain enzyme family (i.e., cytochrome P450 monooxygenases), they were assigned to this family even though the specific gene homolog was not identified.

## 4. Case Study of Atlantic Cod Exposed to Benzo(a)pyrene

**Figure 6:**
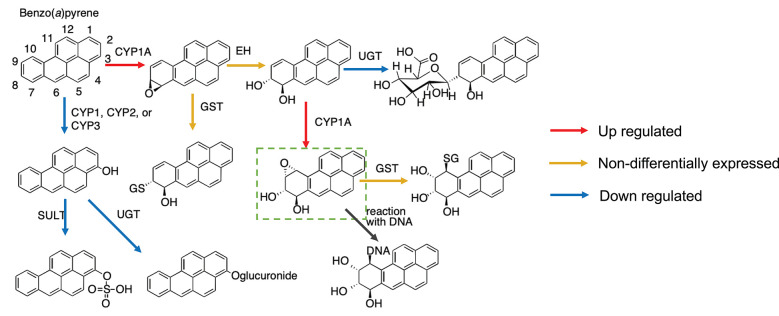
Metabolic pathways of benzo(a)pyrene (BaP) in fish based on Schlenk et al. (2008) and putative changes in cod liver based on differential gene expression after exposure to BaP (Yadetie et al., 2018). The upregulation of cyp1a and down-regulation of sult and ugt expression indicate that the formation of the DNA-reactive metabolite benzo(a)pyrene-7,8-dihydrodiol-9,10-epoxide is preferred.

Mapping gene expression data to the metabolic pathway can give a better understanding of the mechanism by which a toxicant affects the organism. Here, we show a case study of *ex vivo* exposure of Atlantic cod liver slices to benzo(a)pyrene (BaP) (Yadetie et al., 2018). As seen in Figure 5A, all enzymes related to this metabolic pathway were mapped in our model following manual curation. In more details, the metabolic pathway of BaP (adapted from Schlenk et al., 2008) shows that there are two main biotransformation routes of BaP (Figure 6).

Combining the metabolic pathway and gene expression data, we show that the main biotransformation route of BaP in Atlantic cod liver is likely toward the most reactive metabolite, benzo(a)pyrene-7,8-dihydrodiol-9,10-epoxide (Figure 6). Simultaneously, an alternate clearance pathway dependent upon the SULT and UGT enzymes is actually down-regulated, demonstrating clear preference for the primary activation pathway. This shift can be explained by the fact that BaP itself is a well-known ligand for the aryl hydrocarbon receptor (AHR), an important ligand-activated transcription factor regulating the expression of several enzymes in xenobiotic biotransformation pathways, including CYP1A, and recently characterized in cod (Aranguren-Abadía et al., 2019). Although this mechanism is not specific for cod and well described previously (e.g., Kleinow et al., 1998; Schlenk et al., 2008), this demonstrates how a useful insight into evaluation of harmful compounds can be gained by metabolic reconstructions. The expression profiles of the genes can be found in the Supplementary Table S2.

## 5. Discussion and Future Perspectives

In this paper, we presented our experience in reconstructing a draft metabolic network for Atlantic cod liver. As our genome-scale reconstruction work is still on-going, the main intention in this paper is to highlight possible challenges of working on non-model less-annotated organisms. Draft reconstruction for model species is typically the fastest phase in the process, due to the abundance of biochemical knowledge and annotations. However, it becomes less straightforward with a lack of such data. Thanks to enabling computational tools, an alternative is to use existing generic or tissue-specific GEMs that fall within the context of the target reconstruction. In our case, a human liver model was used as a template to generate a draft cod liver model. The fact that it is a well-annotated consensus liver GEM compensated for known physiological and metabolic differences between cod and human, which could be resolved in the next model curation phases.

As demonstrated for the “Metabolism of xenobiotics by cytochrome P450” subsystem, gap filling is an essential part of model curation that need to be applied to all the included subsystems. We survey main gap filling approaches and then describe subsequence reconstruction steps. Several tools were presented to help automate this step. Among those approaches is Meneco (Prigent et al., 2017), a topological gap filling approach, written in Python, that identifies missing reactions through reformulating the gap filling problem as a combinatorial optimization problem. fastGapFill (Thiele et al., 2014) is available in the COBRA toolbox. It selects the set of reactions that optimize a linear score representing the quantitative metabolic production of the metabolic network. Other existing tools can identify missing reactions by integrating taxonomic (Benedict et al., 2014), compartment modularity (Mintz-Oron et al., 2012), or phenotypic knowledge such as experimental flux data (Herrgard and Palsson, 2006; Christian et al., 2009; Kumar and Maranas, 2009; Vitkin and Shlomi, 2012). However, approaches relying on complementary knowledge to perform the gap filling process are not applicable to less-annotated organisms. Accordingly, fastGapFill from the COBRA toolbox could be a suitable choice to automatically fill the gaps in the draft model. Following this step, the refined set of reactions needs to be manually curated since some of them might be added arbitrarily to fulfill selected model criteria, such as model connectivity, by the gap filling tool. The model is then ready for evaluation using flux balance analysis (FBA) (Orth et al., 2010) and phenotype datasets. FBA aims to simulate the cell behavior through finding a set of optimal metabolic fluxes which optimize a metabolic objective, which is often biomass production in bacteria or cancer cells. The metabolic objective of particular cell types in multi-cellular eukaryotes is more difficult to determine however, but methods to infer such objectives have been developed (Bordbar et al., 2020). The simulation results from FBA are compared with experimental values of biomass growth and metabolic fluxes and the model is subjected to refinement until it behaves as desired. The latter allows the reconstructed metabolic network to be analyzed using constraint-based modeling approach. Once those steps are completed, the model can be used to simulate different biological conditions and study the metabolic potential of the organism. For example, it is possible to perform gene knockout studies *in silico* to identify essential genes for various biological conditions using the constraint-based model of the reconstructed network.Thus, insights can be gained into how important physiological processes in the organism, including energy metabolism, growth, and reproductive processes, can be affected by environmental toxicants.

## Data Availability Statement

The datasets generated for this study can be found in online repositories. The names of the repository/repositories and accession number(s) can be found at: https://figshare.com/, doi: 10.6084/m9.figshare.c.5168303.v2.

## Author Contributions

EMH designed the study and wrote the manuscript draft. EMH and XZ performed the *in-silico* work. TF generated the updated Atlantic cod genome assembly. DCZ proposed the idea of the case study and helped XZ with the subsequent analysis and visualization of the results. ME, FY, and AG provided input on all aspects related to biology and environmental toxicology and performed the experimental work of the case study. SF identified and summarized potential tools for gap analysis and helped formatting the manuscript. AG and IJ supervised the work. EMH and XZ worked on the final version of the manuscript which was reviewed and proofread by all authors.

## Conflict of Interest

The authors declare that the research was conducted in the absence of any commercial or financial relationships that could be construed as a potential conflict of interest.
