# Supplementary material for: ReCodLiver0.9: Overcoming Challenges in Genome-Scale Metabolic Reconstruction of a Non-model Species
Source: Front Mol Biosci. 2020 Nov 26;7:591406. doi: 10.3389/fmolb.2020.591406 (PMC7726423; doi:10.3389/fmolb.2020.591406)
Supplement: Supplementary file 1 [file Table_1.pdf]

frontiers

Supplementary Material

Table S1. Summary of reconstruction tools for GEMs of non-model species

Tool	Programming Language	Gap-filling	Databases used for reactions	References
CarveMe	Python	Yes	BIGG Models	(1)
FAME	Python	No	KEGG	(2)
MetaDraft	Python	No	BIGG Models	(3)
Model SEED	Web	Yes	In-house reaction database	(4)
*RAVEN*2.0	Matlab	Yes	KEGG, MetaCyc	(5)

REFERENCES

[1]Machado D, Andrejev S, Tramontano M, Patil KR. Fast automated reconstruction of genome-scale metabolic models for microbial species and communities. Nucleic acids research, 2018;46(15):7542–7553.

[2]Boele J, Olivier BG, Teusink B. FAME, the flux analysis and modeling environment. BMC systems biology, 2012;6(1):8.

[3]Hanemaaijer M, Olivier BG, Röling WF, Bruggeman FJ, Teusink B. Model-based quantification of metabolic interactions from dynamic microbial-community data. PloS one, 2017;12(3).

[4]Henry CS, DeJongh M, Best AA, Frybarger PM, Linsay B, Stevens RL. High-throughput generation, optimization and analysis of genome-scale metabolic models. Nature biotechnology, 2010;28(9):977.

[5]Wang H, Marcišauskas S, Sánchez BJ, Domenzain I, Hermansson D, Agren R, Nielsen J, Kerkhoven EJ. RAVEN 2.0: A versatile toolbox for metabolic network reconstruction and a case study on Streptomyces coelicolor. PLoS computational biology, 2018;14(10):e1006541.
